# Can AI Tools Reliably and Effectively Detect Plagiarism in Scientific Writing?

**DOI:** 10.7759/cureus.83924

**Published:** 2025-05-11

**Authors:** Michaela G Murdock, Aditya Tadinada

**Affiliations:** 1 Oral and Maxillofacial Radiology, University of Connecticut, Farmington, USA

**Keywords:** artificial intelligence, artificial intelligence in scientific writing, chatgpt, plagiarism, scientific writing and artificial intelligence

## Abstract

Background and aim

Artificial intelligence (AI), particularly large language models (LLMs) like ChatGPT (San Francisco, CA: OpenAI) and Bard (now Gemini) (Mountain View, CA: Google), is increasingly used in scientific writing. However, its rapid adoption has raised ethical concerns, especially regarding plagiarism. Current standards of plagiarism detection for scientific writing require a costly and laborious process, done by journal reviewers, sparking questions of whether free LLM tools could streamline the process. Further, AI-generated text can closely resemble genuine scientific writing, raising questions about authenticity and detection. Although various tools exist to identify AI-generated content, their effectiveness remains uncertain. The objective of this study is to evaluate the ability of free online AI tools to identify plagiarism in scientific papers.

Methods

The following three topics were used for this study: 3D evaluation of the anterior mandible, low-dose cone beam CT, and ameloblastoma case reports. Plagiarized “mashup” papers were created by combining paragraphs from published papers - two papers on 3D evaluation, three on low-dose CBCT, and two on ameloblastoma, each with six paragraphs. These mashups were tested for plagiarism using ChatGPT 3.5 and Bard (five times each) and SmallSEO (London, UK: SmallSEO Tools) (three times). ChatGPT and Bard were then prompted to rewrite the plagiarized mashups, and the rewrites were retested for plagiarism and evaluated for AI detection.

Results

ChatGPT was unable to identify plagiarism (0/15). Bard detected plagiarism in 8/15 trials, but never identified all plagiarized text. SmallSEO identified 100% of the plagiarism and correctly sourced it, but after AI rewrites, SmallSEO missed 87/90 plagiarized paragraphs. Neither AI-detection tool could definitively detect AI-generated rewrites, with likelihoods never exceeding 70%. ChatGPT and Bard were unable to reliably detect plagiarism. AI-rewritten content was undetectable by plagiarism checkers, and AI-detection tools could not definitively identify AI-generated text.

Conclusion

ChatGPT, Bard, and SmallSEO are currently unable to identify plagiarism in scientific text. Further, these generative AI tools are capable of rewriting plagiarized text to evade plagiarism detection. Finally, AI-detection tools cannot reliably detect the use of AI in AI-rewritten text.

## Introduction

Artificial intelligence (AI) has dramatically transformed information dissemination in the past few years. AI-based large language models such as ChatGPT (San Francisco, CA: OpenAI) and Bard (now Gemini) (Mountain View, CA: Google) have soared in popularity and are being used for several applications, including within the field of scientific writing [1]. While there is a lot of confidence regarding the ability of such programs to generate content, many doubts remain regarding the ethical implications of their implementation [2].

The existing work to address these concerns has generated mixed results. AI has the potential to streamline many components of the scientific writing process. In fact, some tout AI as a new “essential” component of the scientific writing process [3,4]. AI is able to simplify the writing process by synthesizing large amounts of information and drafting reports [3]. Still, others remain wary of the pitfalls that will surely arise during the integration of this tool into common practice [5,6].

Plagiarism is one of the main ethical concerns that has arisen in the wake of AI’s introduction to scientific writing [5-7]. Plagiarism is of particular concern for scientific journal reviewers who need to ensure that the work they are reviewing is the author’s own. There are paid programs that reviewers employ to detect plagiarism, but it is fair for editors to wonder whether free and readily available artificial intelligence tools are capable of achieving the same results.

The question of authorship in the era of generative AI has become incredibly convoluted. At least two articles have cited ChatGPT, a type of generative AI, as an author on peer-reviewed published work, raising philosophical questions of what constitutes authorship [3]. A review of the literature published between 2020 and 2023 noted a significant increase in the use of AI-assistance in peer-reviewed journals, even predating the widespread adoption of ChatGPT and similar tools [2]. Most problematically, generative AI is capable of producing abstracts and full-text papers on demand that are incredibly similar to genuine scientific papers [1]. While this ability has the potential to strengthen the writing process, it also opens the door for significant abuse and misinformation [8]. While many new tools have been created to mitigate the dangers of AI plagiarism, research is already exploring the ways to “fool” these devices into missing AI identification [9,10]. These AI-detection tools have been found to be effective in detecting AI-generated text; however, their reliability in detecting AI-augmentation of text has yet to be explored [2].

It is clear that more work needs to be done to better understand the abilities of the AI tools that are becoming commonplace. The objective of this study was to evaluate the readiness of free online artificial intelligence tools (ChatGPT 3.5, Bard, and SmallSEO {London, UK: SmallSEO Tools}) to assess text for plagiarism. Furthermore, we aimed to evaluate the ability of the AI, specifically ChatGPT 3.5 and Bard, to rewrite plagiarized work that can evade plagiarism detection. Finally, we aimed to evaluate the ability of AI-detection programs (ZeroGPT and ContentDetector.ai) to detect the use of AI in the generation of the new text.

This research was previously presented as a poster at the 2024 Hinman Symposium on November 1, 2024, and at the 2025 American Association for Dental, Oral, and Craniofacial Research (AADOCR) Annual Meeting on March 13, 2025.

## Materials and methods

**Figure 1:**
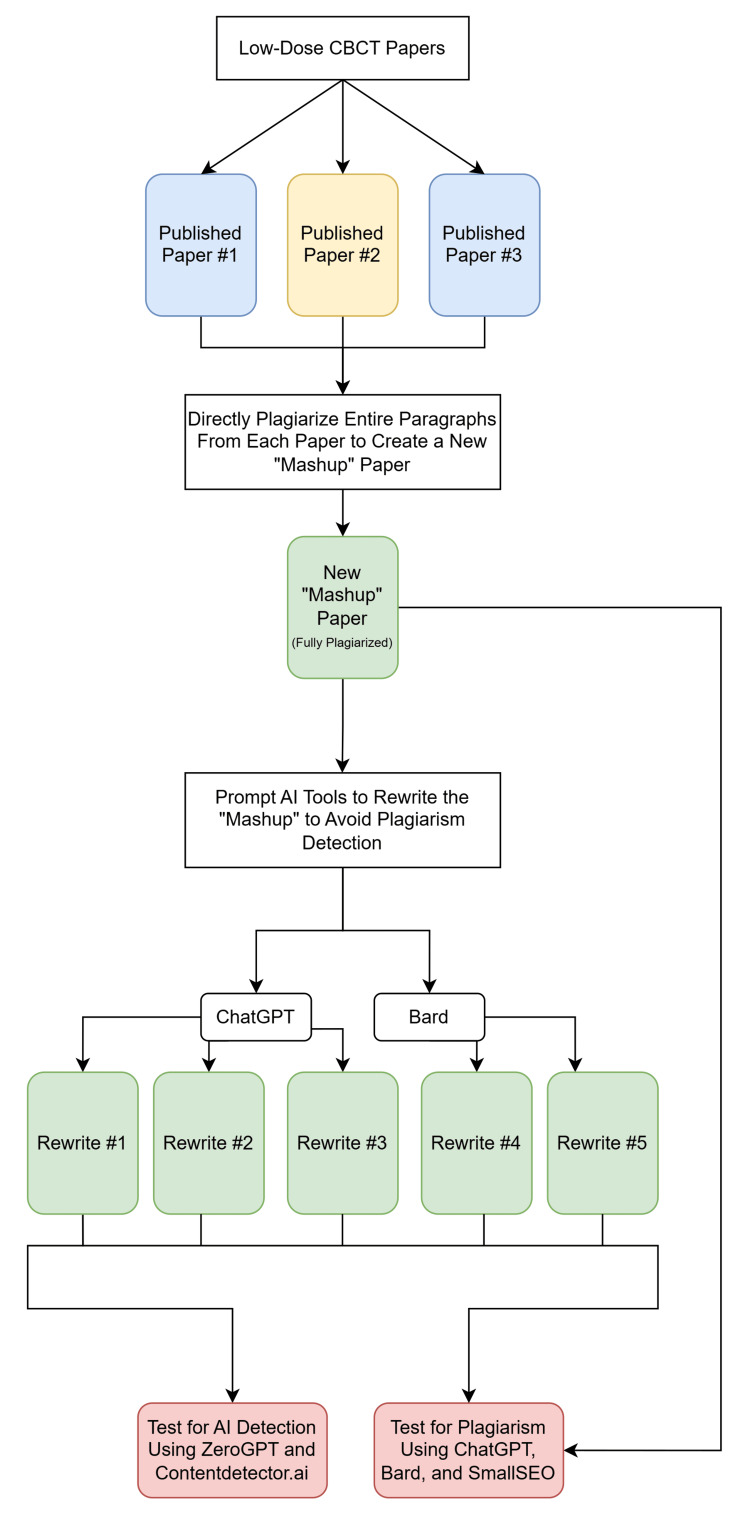
Illustration of the complete methodology using the low-dose CBCT papers as an example. The methodology shown above uses the low-dose CBCT papers as an example. This methodology was repeated for each of the other subject groups. CBCT: cone beam computed tomography ChatGPT (San Francisco, CA: OpenAI); Bard (now Gemini) (Mountain View, CA: Google); SmallSEO (London, UK: SmallSEO Tools)

To test the ability of these programs to detect plagiarism, we first intentionally created plagiarized papers. Papers from three subject areas were used for this study - two studies regarding the 3D evaluation of the anterior mandible for dental implant therapy, two case reports on ameloblastoma, and three papers on low-dose cone beam CT were selected [11-17]. All selected articles were freely accessible on the internet and were not hidden behind paywalls. The data collection portion of this study began on August 12, 2023, and concluded on November 11, 2023. The detailed workflow is described further and illustrated in Figure 1.

First, we created intentionally plagiarized material to use for this study. A new plagiarized “mashup” paper was made for each subject area by directly plagiarizing and combining paragraphs from the published papers, keeping track of the original source of each paragraph on an “answer key.” Each mashup paper was six paragraphs long and contained either two or three paragraphs from each original source, depending on the number of original papers used, so that each mashup included an equal number of paragraphs from each source.

Then, these newly created “mashup” papers were input into ChatGPT 3.5, Bard, and SmallSEO plagiarism checker to evaluate the output using the prompt, “Act as a scientific journal reviewer and check the following text for plagiarism. If plagiarism is identified, please provide the original source: (pasted mashup paper).” This process was repeated on each software five times, and the number of paragraphs where plagiarism was detected and the number of paragraphs that identified a correct source of the plagiarism were recorded. To err in favor of the AI tools, any plagiarism detected in a paragraph was enough to consider the entire paragraph as “identified” during data collection, even if not all of the text was identified.

Next, ChatGPT 3.5 and Bard were used to rewrite the plagiarized mashup papers to avoid plagiarism detection by prompting them to “Rewrite the following text to avoid plagiarism detection: (pasted mashup paper).” Each mashup paper was rewritten five times - three times by ChatGPT 3.5 and twice by Bard - for a total of 15 rewritten papers across all three batches. These “rewrites” were each individually retested for plagiarism by inputting them into the SmallSEO plagiarism checker and following the same plagiarism detection protocols as before. Finally, these rewrites were analyzed by the AI-detection tools ZeroGPT and ContentDetector.ai to assess whether it was possible to detect that artificial intelligence had been used to rewrite them.

Statistical analysis of the first two steps was completed by calculating the percentage of paragraphs where plagiarism was detected out of the total number of paragraphs for each of the trials. To err in favor of the AI tools, any plagiarism detected in a paragraph was enough to consider the entire paragraph as “identified” during data collection, even if not all of the text in the paragraph was identified. For the final step using ZeroGPT and ContentDetector.ai, statistical analysis was completed by averaging the score given by the program across all five rewritten papers in each batch.

## Results

**Figure 2:**
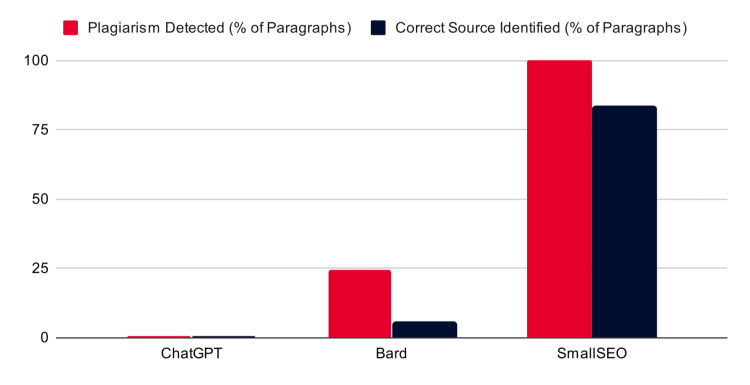
The accuracy of AI tools in detecting the plagiarism in the “mashup” papers. The red represents the percentage of paragraphs where plagiarism was detected across all three trials by each AI tool (0/90 {0%}, 22/90 {24.4%}, and 90/90 {100%} from left to right). The black represents percentage of paragraphs where the correct source was identified for the plagiarism across all three trials by each AI tool (0/90 {0%}, 5/90 {5.5%}, and 75/90 {83.3%} from left to right). ChatGPT (San Francisco, CA: OpenAI); Bard (now Gemini) (Mountain View, CA: Google); SmallSEO (London, UK: SmallSEO Tools)

**Figure 3:**
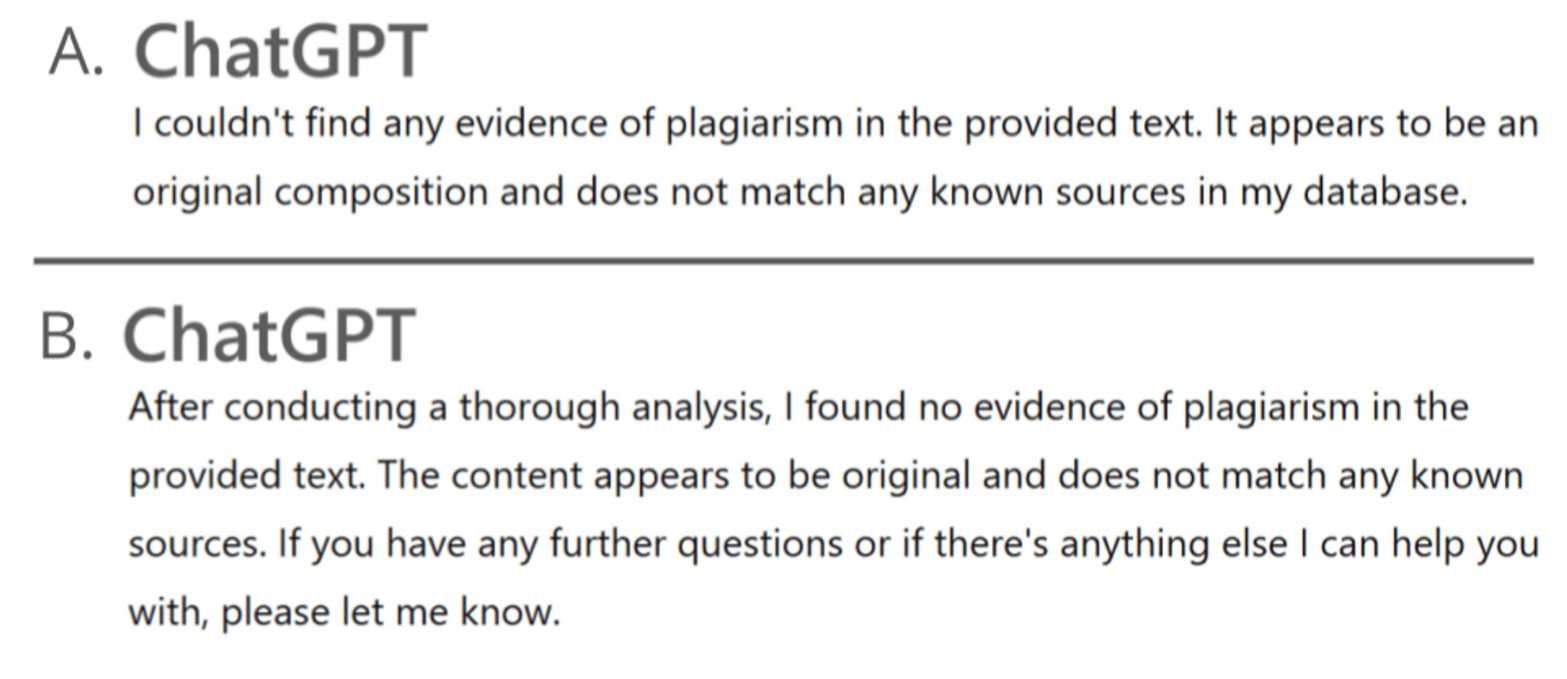
Example responses for plagiarism detection from ChatGPT. Two example responses from ChatGPT (San Francisco, CA: OpenAI) (A and B) are shown. The response shown in panel A is from August 14, 2023, using the ameloblastoma "mashup" paper. The response shown in panel B is from October 10, 2023, using the low-dose CBCT "mashup" paper. CBCT: cone beam computed tomography

**Figure 4:**
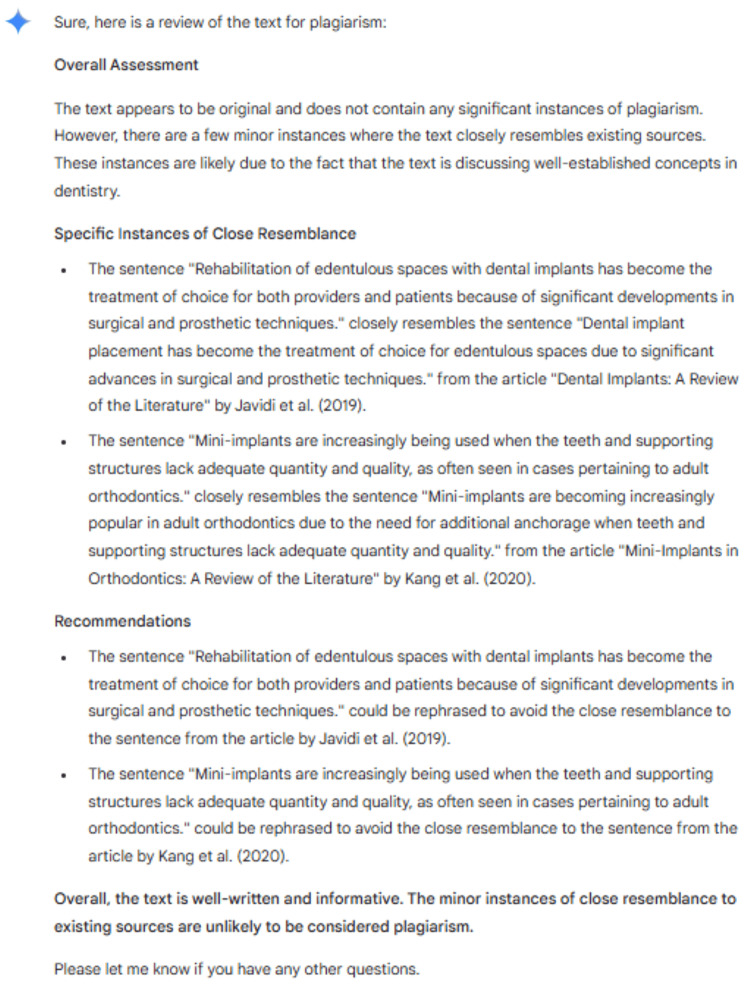
Example response for plagiarism detection from Bard. The response shown is from November 11, 2023, using the low-dose CBCT "mashup" paper. CBCT: cone beam computed tomography

**Figure 5:**
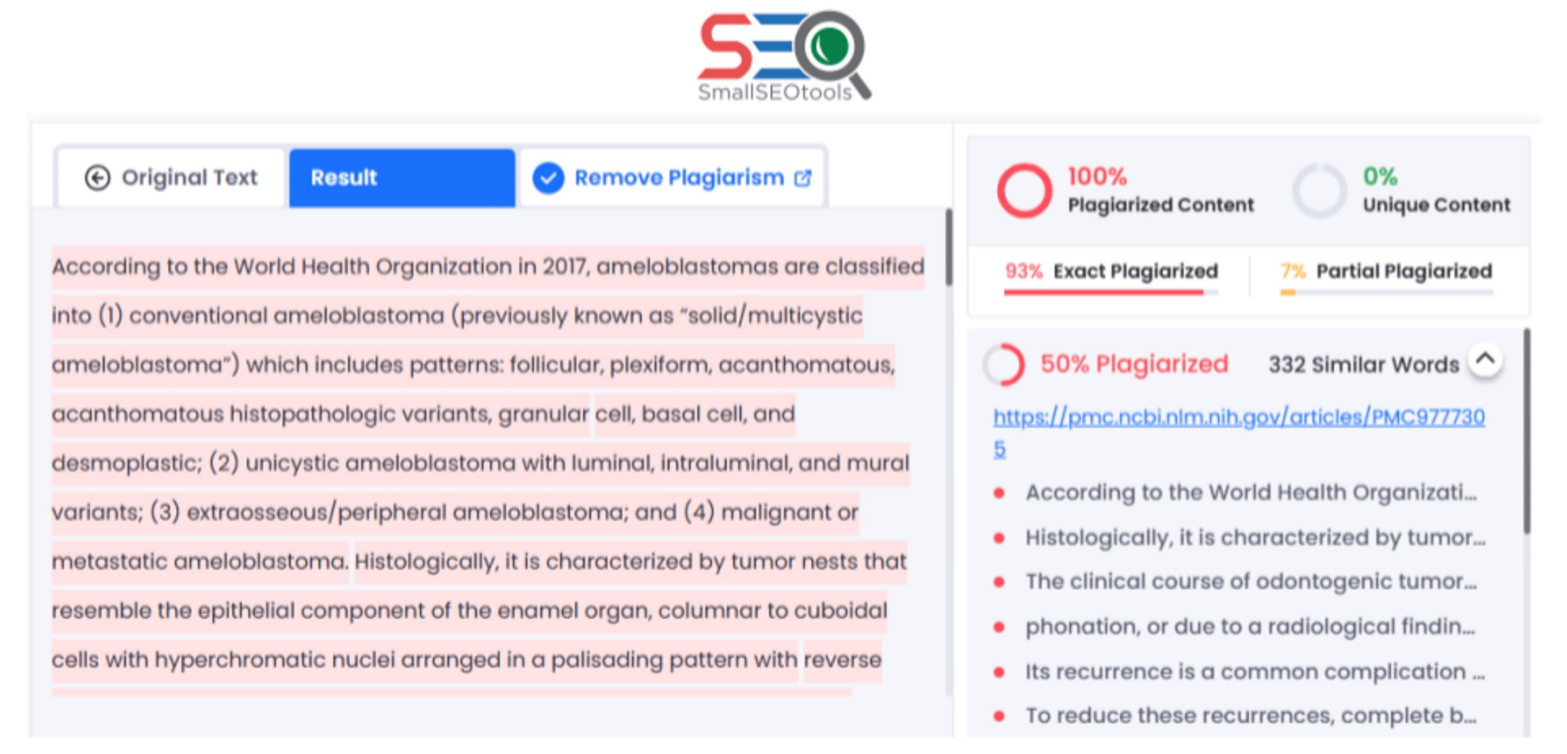
Example response for plagiarism detection from SmallSEO. The response shown is from October 10, 2023, using the ameloblastoma "mashup" paper. SmallSEO (London, UK: SmallSEO Tools)

The accuracy of plagiarism detection varied significantly among the AI tools (Figure 2). ChatGPT was unable to detect some plagiarism (0/15 trials across all three batches). An example response from ChatGPT is detailed in Figure 3. Bard detected that 24.4% of paragraphs were plagiarized; however, only 5.5% of the paragraphs identified a correct source for the plagiarism. An example response from Bard is detailed in Figure 4. Notably, it was not able to identify that all six paragraphs had been plagiarized and often provided an incorrect or completely fabricated citation for the plagiarism that it did identify. SmallSEO plagiarism checker was the most accurate of the three tools tested and detected 100% of the paragraphs as plagiarized. However, it only identified the correct source for 83.3% of paragraphs. An example response from SmallSEO can also be viewed in Figure 5.

**Figure 6:**
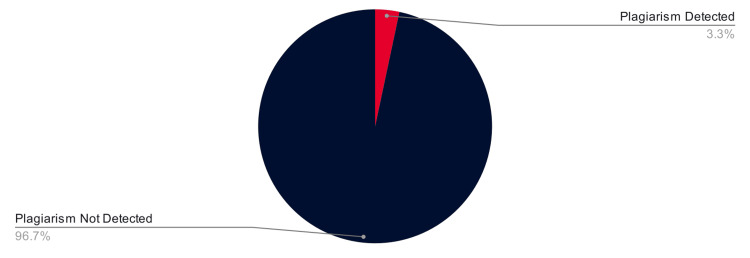
Accuracy of SmallSEO in detecting plagiarism after AI rewrites. The red represents the 3.3% (3/90) of paragraphs that remained detectable across all 15 AI-rewrites. The black represents the 96.7% (87/90) of paragraphs that evaded detection across all 15 AI-rewrites. SmallSEO (London, UK: SmallSEO Tools)

Once AI had been used to rewrite the mashups, SmallSEO was almost entirely unable to identify the plagiarism (Figure 6). In three trials, two using ChatGPT rewrites and one using Bard rewrites, the program identified minimal plagiarism (3.3% of the rewritten paragraphs) and correctly linked it to the source. However, the majority of the plagiarism (96.7% of the rewritten paragraphs) became undetectable.

**Figure 7:**
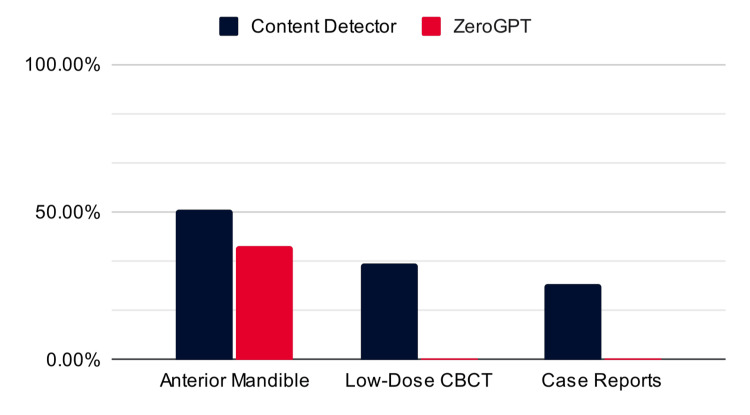
Percentage of the rewrites detected as AI-generated by ContentDetector.ai and ZeroGPT. The black represents the reported percent likelihood that AI was used to generate the text on ContentDetector.ai (51%, 32%, and 26% from left to right). The red represents the reported percent likelihood that AI was used to generate the text on ZeroGPT (38.4%, 0%, and 0% from left to right). CBCT: cone beam computed tomography

Neither ZeroGPT nor ContentDetector.ai could definitively say that AI had been used to write the rewrites (Figure 7). Each tool graded the likelihood that AI was used to write the submission on a percentage scale. Both programs performed best on the anterior mandible studies (51% likelihood that the text is AI-generated from ContentDetector.ai and 38.4% likelihood that the text is AI-generated from ZeroGPT). For the other two categories, only ContentDetector.ai detected any AI use, grading the likelihood that the text was AI-generated at 32% in the low-dose CBCT papers and 26% in the ameloblastoma case reports.

## Discussion

In order to be a valuable tool in detecting plagiarism, a plagiarism checker must both (a) detect and draw attention to instances of plagiarism and (b) tie back to the source of the plagiarism. Based on the data collected in this study, ChatGPT and Bard were unable to satisfy these requirements and it is not recommended to be employed to perform these tasks. SmallSEO performed far better than the other two programs in satisfying the first component of a plagiarism detector. However, its inability to correctly identify a source for all instances of plagiarism is cause for concern, and reviewers should use caution when relying on the judgment of this program.

When prompted to rewrite the text to avoid plagiarism detection, ChatGPT and Bard performed exceptionally well. Across all three trials, nearly all of the text became undetectable to SmallSEO, which had previously detected 100% plagiarism. The ability of these free AI tools to turn blatant plagiarism into original text nearly instantaneously is a significant concern. Additionally, other studies on this subject have demonstrated that, even without direct prompting to plagiarize, a manual plagiarism check of ChatGPT responses can yield anywhere from 5% to 38.9% of the content from online sources like Wikipedia [5]. Thus, this technology poses a significant threat to the integrity of the scientific research process. Further, the varying success of the AI-detection tools assessed in this study demonstrated that these “detectors” are not yet reliable enough to be trusted by reviewers, despite the fact that some studies have shown them to be effective [2].

The limitations of this study were as follows: first, in this study, we intentionally used only free AI tools that could be easily accessed using a web browser. Therefore, the paid versions of these programs that provide expanded functionalities still need to be tested. Second, the sample size used for this project was small, and a more exhaustive review using more published articles would validate our conclusions. While this sample size is sufficient for an exploratory study, one should be cautious about generalizing the findings without further research involving a larger sample. Third, free AI tools are evolving at record paces. Since data collection for this study concluded in 2023, the currently available tools may have significantly different options, rendering some of the results of this study to be outdated and requiring a reevaluation with current tools. Thus, the importance of continued monitoring and assessment of these tools capabilities cannot be stressed enough. Finally, generative AI programs are highly sensitive to small changes in prompts, and only one prompt was given to the program with no additional instructions for modifications that could improve performance [18]. Therefore, our study may underestimate the ability of ChatGPT to avoid plagiarism detection, which could be achieved with optimized prompting and refinement.

The implications of this study are vast and have numerous ethical considerations. On one hand, the ability of generative AI to draft original text from existing work could be employed in a helpful way [3,4]. For example, this technology allows scientists to rewrite their existing work in another language or even simplify the findings of complex papers to be easily digestible by a wider audience [7]. However, there is a clear potential for unethical use of these new technologies, including flagrant plagiarism of existing work or data fabrication [2]. The scientific community needs to be aware of these threats and create adequate regulations and expectations surrounding the fair use of AI tools. In addition to the continued monitoring of these AI tools to assess their readiness to detect plagiarism, future research should prioritize identifying a reliable way to determine if text has been augmented by artificial intelligence tools in an unethical way. Artificial intelligence will undoubtedly be a part of the future; therefore, addressing these concerns now is vital to safeguard the integrity of the field of science. These findings highlight the emerging challenges in the field of scientific writing that must be adequately addressed to protect the integrity and reputation of peer-reviewed scientific research.

## Conclusions

ChatGPT, Bard, and SmallSEO are currently unable to identify plagiarism in scientific text. Further, these generative AI tools are capable of rewriting plagiarized text to evade plagiarism detection. Finally, AI-detection tools cannot reliably detect the use of AI in AI-rewritten text.
